# Clozapine treatment and risk of COVID-19 infection: retrospective cohort study

**DOI:** 10.1192/bjp.2020.151

**Published:** 2020-07-27

**Authors:** Risha Govind, Daniela Fonseca de Freitas, Megan Pritchard, Richard D. Hayes, James H. MacCabe

**Affiliations:** 1 Institute of Psychiatry, Psychology and Neuroscience, King's College London; and National Institute for Health Research (NIHR) Biomedical Research Centre, South London and Maudsley NHS Foundation Trust and King's College London, UK; 2 Institute of Psychiatry, Psychology and Neuroscience, King's College London; and National Institute for Health Research (NIHR) Biomedical Research Centre, South London and Maudsley NHS Foundation Trust and King's College London; and National Psychosis Unit, South London and Maudsley NHS Foundation Trust, London, UK

**Keywords:** COVID-19, clozapine, antipsychotics, epidemiology, psychotic disorders

## Abstract

**Background:**

Clozapine, an antipsychotic with unique efficacy in treatment-resistant psychosis, is associated with increased susceptibility to infection, including pneumonia.

**Aims:**

To investigate associations between clozapine treatment and increased risk of COVID-19 infection in patients with schizophrenia-spectrum disorders who are receiving antipsychotic medications in a geographically defined population in London, UK.

**Method:**

Using information from South London and Maudsley NHS Foundation Trust (SLAM) clinical records, via the Clinical Record Interactive Search system, we identified 6309 individuals who had an ICD-10 diagnosis of schizophrenia-spectrum disorders and were taking antipsychotics at the time of the COVID-19 pandemic onset in the UK. People who were on clozapine treatment were compared with those on any other antipsychotic treatment for risk of contracting COVID-19 between 1 March and 18 May 2020. We tested associations between clozapine treatment and COVID-19 infection, adjusting for gender, age, ethnicity, body mass index (BMI), smoking status and SLAM service use.

**Results:**

Of 6309 participants, 102 tested positive for COVID-19. Individuals who were on clozapine had increased risk of COVID-19 infection compared with those who were on other antipsychotic medication (unadjusted hazard ratio HR = 2.62, 95% CI 1.73–3.96), which was attenuated after adjusting for potential confounders, including clinical contact (adjusted HR = 1.76, 95% CI 1.14–2.72).

**Conclusions:**

These findings provide support for the hypothesis that clozapine treatment is associated with an increased risk of COVID-19 infection. Further research will be needed in other samples to confirm this association. Potential clinical implications are discussed.

Clozapine is an antipsychotic with unique efficacy in treatment-resistant psychosis and, for many people, it is the only effective treatment.^1^ It is associated with a reduction in hospital admissions, overall mortality and suicide risk in schizophrenia.^2–5^ People with schizophrenia have an increased mortality compared with the general population.^6,7^ Some of this excess mortality is attributable to pneumonia^8–11^ and much of this increase may be attributable to higher rates of smoking.^12^ However, there appears to be an additional effect of clozapine treatment.^13–16^ In the study of Kuo and colleagues, treatment with clozapine was associated with approximately a doubling of the risk of pneumonia.^14^ However, confounding by indication could have affected these results: clozapine is prescribed to people with treatment-resistant schizophrenia, and such individuals are likely to have a range of comorbidities that increase the risk of infection, such as smoking and other substance misuse, poor diet and a sedentary lifestyle.^17^ It is also plausible that some of the adverse effects of clozapine, such as diabetes, weight gain and hypersalivation (leading to aspiration pneumonia^18^), could lie on the causal pathway between clozapine treatment and the risk of infection. Clozapine treatment appears to have multiple effects on the innate immune system, including transient eosinophilia, cytokine release and fever during early treatment, and neutropaenia and agranulocytosis in a small minority.^19^ There is emerging evidence that adaptive immunity is also affected by clozapine,^20^ with a reduction in all three classes of circulating immunoglobulins (IgM, IgA and IgG) in clozapine-treated patients compared with those on other antipsychotics. COVID-19 is a novel infection caused by SARS-Cov-2, causing pneumonia in severe cases. It arose in China in late 2019 and was declared a global pandemic by the World Health Organization (WHO) in March 2020.^21^ Given the effects of clozapine on immunity and the increased risk of pneumonia, we investigated whether clozapine treatment was associated with an increased risk of COVID-19 infection in patients with schizophrenia and other psychoses treated with antipsychotics in a geographically defined population in London during the COVID-19 pandemic.

## Method

### Setting and ethics statement

This retrospective cohort study used data from the South London and Maudsley NHS Foundation Trust (SLAM), one of Europe's largest secondary mental healthcare providers. In the UK, mental health services are provided on the basis of defined geographical catchment areas under the National Health Service (NHS). SLAM provides all aspects of secondary mental healthcare to over 1.3 million people of four London boroughs (Lambeth, Southwark, Lewisham and Croydon). From 2006, SLAM has used a fully electronic health records system and the Clinical Records Interactive Search system (CRIS), supported by the National Institute for Health Research (NIHR) Specialist Biomedical Research Centre for Mental Health. CRIS was established in 2008 to enable researchers to search and retrieve de-identified clinical records from SLAM. The protocol for CRIS has been described in detail in an open-access publication.^22^ CRIS was approved as an anonymised data resource for secondary analysis by Oxfordshire Research Ethics Committee C (reference 18/SC/0372). The data linkage to King's College Hospital for admissions regarding COVID-19 infections took place under Regulation 3(2) and Regulation 3(3) of the Health Service Control of Patient Information Regulations 2002 (COPI).

### Analytical cohort and data extraction

The cohort comprised individuals who fulfilled all three of the following inclusion criteria: ICD-10 diagnosis of schizophrenia-spectrum disorders (F2*); taking antipsychotic medication between 1 December 2019 and 1 March 2020; and receiving out-patient or in-patient care at SLAM on 1 March 2020. This date was chosen because it was before 12 March 2020, the date of the first diagnosed case of COVID-19 in SLAM, so there was no risk of reverse causation (the presence of COVID-19 infection affecting the exposures).

SQL Server Management Studio version 15.0 for Windows was used to extract the data. The day of data extraction was 18 May 2020. The index period, from which medication data were gathered, was from 1 December 2019 to 1 March 2020. Patients were followed up from 1 March 2020 until they were diagnosed COVID-19 positive, died or reached the end of the observation period (18 May 2020), whichever occurred first.

Even though specific structured fields exist within CRIS, these are often incomplete and much of the useful information in CRIS is within the free-text fields of clinical notes. To fully exploit this database, data from structured fields are augmented by data extracted from free-text fields of clinical records, using custom-built natural language processing (NLP) algorithms.^23^ NLP algorithms are able to outperform keyword searches because they take into account the linguistic context of terms of interest, for example temporal modifiers (e.g. ‘on clozapine’ versus ‘previously took clozapine’). Data from four NLP algorithms were used in this study: diagnosis, medication, smoking and body mass index (BMI).

The diagnosis algorithm was used for the inclusion criteria to identify individuals who were ever diagnosed with ICD-10 schizophrenia-spectrum disorders (F2*). The precision and recall scores for the diagnosis algorithm are 100% and 65% respectively.^23^

The medication algorithm data were also used for the inclusion criteria to identify individuals who were on an antipsychotic medication between 1 December 2019 and 1 March 2020, the index period. This algorithm provides specific results for antipsychotic medication. The antipsychotic prescriptions included in this analysis were clozapine, olanzapine, risperidone, aripiprazole, amisulpride, paliperidone, flupentixol, haloperidol, zuclopenthixol, quetiapine, fluphenazine, sulpiride, lurasidone, trifluoperazine, chlorpromazine, pipotiazine, penfluridol, droperidol, pimozide, thioridazine, promazine, ziprasidone hydrochloride, levomepromazine and pericyazine. The precision and recall scores for the antipsychotics part of the medication algorithm are 88% and 90% respectively.^23^

The smoking algorithm was used to identify the smoking status of each patient. The ‘current smoker’ status was based on data recorded between 1 March 2019 and 1 March 2020. The ‘past smoker’ and ‘never smoked’ statuses were based on all available information in the electronic health record. In the underlying patient records, smoking status may be recorded repeatedly, i.e. each time this information is entered into the patient record. Consequently, for some patients, the smoking algorithm may identify more than one smoking status per patient. Where this was the case, we took the highest smoking status in the hierarchy ‘current smoker’ > ‘past smoker’ > ‘never smoked’. The precision (P) and recall (R) scores for each status of the smoking algorithm are as follows: for ‘current smoker’ status, *P* = 79% and *R* = 87%; for ‘past smoker’ status, *P* = 68% and *R* = 38%; for ‘never smoked’ status, *P* = 72% and *R* = 75%.^23^

The BMI algorithm was used to extract the most recent BMI measurement for each patient in the entire patient record. To exclude erroneous values from the results of this algorithm, we rejected values outside the range 15–70 kg/m^2^. The overall precision and recall scores for the BMI algorithm are 89% and 78% respectively.^23^

All the NLP algorithm outputs were also supplemented by the data in the structured fields, data in the health records (such as data from ICD-10 diagnosis forms for diagnosis data) and pharmacy dispensary data for medication data.

Of all patients in SLAM, 6309 met the inclusion criteria of individuals with ICD-10 diagnoses of schizophrenia-spectrum disorders (F2*) who were on antipsychotic medication during the index period.

### Main outcome measure

The outcome of interest was infection with COVID-19 during the follow-up period (1 March to 18 May 2020). These data were collated by combining information from the SLAM pathology laboratory results, the presence of a clinician-entered alert on SLAM records reading ‘COVID-19 positive’ and information provided by local general hospitals (King's College Hospital and Princess Royal University Hospital) for COVID-19-related admissions.

### Exposure of interest and potential confounding variables

In keeping with the cohort study design, the exposure of interest and potential confounders were recorded before the start of follow-up. People who were on clozapine treatment at any time between 1 December 2019 and 1 March 2020 were designated as the exposed group. Those on any type or combination of antipsychotic treatment that did not include clozapine during this time constituted the unexposed group.

We considered the following potential confounders: sociodemographic characteristics, health and use of SLAM services. The sociodemographic information was age, gender and ethnicity. The health information was smoking status and BMI. The SLAM services use information comprised data on whether the person was an in-patient on 1 March 2020 and the number of days they were in contact with the SLAM services between 1 December 2019 and 1 March 2020. The contact with SLAM services included any form of in-patient and out-patient communication, such as email or phone or face-to-face consultations.

### Statistical analysis

The data were analysed using STATA for Windows version 15.1. Using Cox proportional hazard models, we calculated hazard ratios for COVID-19-positive status in clozapine-treated participants compared with those treated with other antipsychotics. We censored observations at the date of death, date of COVID-19-positive test or 18 May 2020, whichever occurred first. We confirmed that the data satisfied the proportional-hazards assumptions using Schoenfeld residuals.

Three potential confounding variables contained missing data: smoking, ethnicity and BMI. First, we analysed the entire cohort using only variables with no missing data. Then, in a complete case analysis, we ran the same analyses, excluding individuals for whom there were any missing data across any of the exposures investigated (*n* = 5535). Since the results were very similar, we were confident that the complete case analysis was unlikely to suffer from undue selection bias. We present results from the complete case analysis and the results of the whole-cohort analysis in the supplementary material available at https://doi.org/10.1192/bjp.2020.151.

Crude and adjusted models were constructed, first controlling for age, gender and ethnicity; then controlling for age, gender, ethnicity, in-patient status and number of contact days with the SLAM services. Last, we constructed a fully adjusted model controlling for all variables: age, gender, ethnicity, in-patient status, number of contact days with the SLAM services, smoking status and BMI. All models were built using data from the 5535 individuals with complete data. The above models were repeated using the whole cohort (*n* = 6309) without including the variables with missing data (ethnicity, smoking status, BMI): these results are in supplementary Table 1.

## Results

There were 6309 active patients with schizophrenia-spectrum disorders (F2*) who were receiving any type of antipsychotic treatment during the beginning of the follow-up period. The sample mean age was 46.5 years (s.d. = 14.8) and men account for 61.7% of the sample. The sample's ethnic description is: 33.2% White (including White British, Irish or any other White background), 50.6% Black (including Black African, Black Caribbean, Other Black background, White and African, and White and Caribbean), 13.7% any Asian and Other ethnic background; 2.5% had missing data on ethnicity.

Table 1 summarises the demographic features of all the SLAM patients who qualified for the inclusion criteria (*n* = 6309). Of the individuals who were on clozapine, 66% were male, 46% were Black, 80% were current smokers and 48% had high BMI (obese). Compared with participants not on clozapine treatment, a higher proportion of clozapine-treated participants were in-patients in the hospital on 1 March 2020 (13 *v.* 6%), and clozapine-treated participants had more contact days with the SLAM services in the previous 3 months.

**Table 1:** Sample characteristics of all SLAM patients who qualified for the inclusion criteria, presented according to those who were and were not on clozapine treatment

	Not on clozapine treatment, *n* (%)	On clozapine treatment, *n* (%)
Total (*n* = 6309)	5027 (79.68)	1282 (20.32)
Gender		
Male	3047 (60.61)	847 (66.07)
Female	1980 (39.39)	435 (33.93)
Age		
<29 years	817 (16.25)	131 (10.22)
30–39 years	1055 (20.99)	281 (21.92)
40–49 years	1082 (21.52)	338 (26.37)
50–59 years	1145 (22.78)	369 (28.78)
60–69 years	546 (10.86)	127 (9.91)
≥70 years	382 (7.60)	36 (2.81)
Ethnicity		
White	1579 (31.41)	516 (40.25)
Black	2607 (51.86)	586 (45.71)
Asian, Other and Not stated	691 (13.75)	170 (13.26)
Missing	150 (2.98)	10 (0.78)
In-patient on the first day of follow-up period^a^		
No	4735 (94.19)	1113 (86.82)
Yes	292 (5.81)	169 (13.18)
SLAM service contact during index period^b^		
<4 days	1902 (37.84)	242 (18.88)
4–7 days	1575 (31.33)	479 (37.36)
≥8 days	1550 (30.83)	561 (43.76)
Smoking status		
Current smoker	3154 (62.74)	1028 (80.19)
Past smoker	1457 (28.98)	232 (18.10)
Never smoked	326 (6.48)	18 (1.40)
Missing	90 (1.79)	4 (0.31)
BMI		
Underweight and healthy	1601 (31.85)	264 (20.59)
Overweight	1335 (26.56)	358 (27.93)
Obese	1499 (29.82)	610 (47.58)
Missing	592 (11.78)	50 (3.90)

SLAM, South London and Maudsley NHS Foundation Trust; BMI, body mass index.

a. First date of follow period is 1 March 2020.

b. Index period is between 1 December 2019 and 1 March 2020, which is 3 months prior to the follow-up period.

Table 2 summarises the demographic features presented according to their outcome status: COVID-19 positive or not COVID-19 positive. Of those who were COVID-19 positive, 41% were receiving clozapine treatment, whereas of those who were not COVID-19 positive, only 20% were receiving clozapine treatment. A higher proportion of COVID-19-positive patients were in-patients and COVID-19-positive patients had more contact days with the SLAM services.

**Table 2:** Sample characteristics of all SLAM patients who qualified for the inclusion criteria, presented according to those who tested positive for COVID-19 and those who did not during the follow-up period (1 March to 18 May 2020 inclusive)

	Not COVID-19 positive, *n* (%)	COVID-19 positive, *n* (%)
Total sample (*n* = 6309)	6207 (98.38)	102 (1.62)
On clozapine treatment		
No	4967 (80.02)	60 (58.82)
Yes	1240 (19.98)	42 (41.18)
Gender		
Male	3838 (61.83)	56 (54.90)
Female	2369 (38.17)	46 (45.10)
Age		
<29 years	937 (15.10)	11 (10.78)
30–39 years	1315 (21.19)	21 (20.59)
40–49 years	1408 (22.68)	12 (11.76)
50–59 years	1485 (23.92)	29 (28.43)
60–69 years	657 (10.58)	16 (15.69)
≥70 years	405 (6.52)	13 (12.75)
Ethnicity		
White	2069 (33.33)	26 (25.49)
Black	3129 (50.41)	64 (62.75)
Asian, Other and Not stated	853 (13.74)	8 (7.84)
Missing	156 (2.51)	4 (3.92)
In-patient on the first day of follow-up period^a^		
No	5804 (93.51)	44 (43.14)
Yes	403 (6.49)	58 (56.86)
SLAM service contact during index period^b^		
<4 days	2137 (34.43)	7 (6.86)
4–7 days	2035 (32.79)	19 (18.63)
≥8 days	2035 (32.79))	76 (74.51)
Smoking status		
Current smoker	4104 (66.12)	78 (76.47)
Past smoker	1669 (26.89)	20 (19.61)
Never smoked	342 (5.51)	2 (1.96)
Missing	92 (1.48)	2 (1.96)
BMI		
Underweight and healthy	1843 (29.69)	22 (21.57)
Overweight	1675 (26.99)	18 (17.65)
Obese	2054 (33.09)	55 (53.92)
Missing	635 (10.23)	7 (6.86)

SLAM, South London and Maudsley NHS Foundation Trust; BMI, body mass index.

a. First date of follow period is 1 March 2020.

b. Index period is between 1 December 2019 and 1 March 2020, which is 3 months prior to the follow-up period.

The Cox regression analysis was performed with data of the 5535 individuals with complete information (774 participants were excluded because of missing data: Table 1), and the mean follow-up period was 78.00 days (s.d. = 7.03). Of these 5535 individuals, 92 tested positive for infection with COVID-19 during the follow-up period. Table 3 shows the hazard ratios for COVID-19 infection associated with being on clozapine-treatment in the crude and adjusted models. The crude model shows a hazard ratio of 2.62 (95% CI 1.73–3.96) for participants receiving clozapine treatment and COVID-19 positive. This increased to 3.06 (95% CI 2.01–4.67) after adjusting for sociodemographic factors (age, gender, ethnicity). It was attenuated to 1.85 (95% CI 1.20–2.85) after adjusting for in-patient status and SLAM service contact. It was further attenuated to 1.76 (95% CI 1.14–2.72) after adjusting for BMI and smoking status.

**Table 3:** Multivariate Cox analysis of association between receiving clozapine treatment and COVID-19 infection between 1 March and 18 May 2020 inclusive in 5535 individuals (92 COVID-19 positive)

Risk factors	HR (95% CI)	HR (95% CI)	HR (95% CI)	HR (95% CI)
On clozapine treatment				
No	1.00	1.00	1.00	1.00
Yes	2.62 (1.73–3.96)	3.06 (2.01–4.67)	1.85 (1.20–2.85)	1.76 (1.14–2.72)
Gender				
Male		1.00	1.00	1.00
Female		1.30 (0.86–1.97)	1.43 (0.94–2.17)	1.32 (0.86–2.04)
Age				
<29 years		1.00	1.00	1.00
30–39 years		1.03 (0.49–2.18)	1.25 (0.59–2.66)	1.14 (0.53–2.42)
40–49 years		0.54 (0.23–1.27)	0.69 (0.29–1.63)	0.64 (0.27–1.52)
50–59 years		1.30 (0.64–2.64)	1.95 (0.95–3.98)	1.78 (0.87–3.64)
60–69 years		1.73 (0.78–3.83)	2.88 (1.29–6.45)	2.76 (1.22–6.23)
≥70 years		3.31 (1.43–7.66)	4.10 (1.76–9.51)	4.35 (1.85–10.26)
Ethnicity				
White		1.00	1.00	1.00
Black		1.98 (1.23–3.19)	1.81 (1.12–2.91)	1.70 (1.04–2.77)
Asian, Other and Not stated		0.74 (0.30–1.82)	0.75 (0.31–1.84)	0.78 (0.32–1.92)
In-patient on the first day of follow-up period^a^				
No			1.00	1.00
Yes			10.31 (6.01–17.67)	10.08 (5.86–17.34)
SLAM service contact over index period^b^				
<4 days			1.00	1.00
4–7 days			2.58 (1.02–6.53)	2.62 (1.03–6.65)
≥8 days			3.56 (1.41–9.01)	3.64 (1.42–9.30)
Smoking status				
Current smoker				1.00
Past smoker				1.06 (0.62–1.81)
never smoked				0.69 (0.16–2.88)
BMI				
Underweight and healthy				1.00
Overweight				0.93 (0.49–1.75)
Obese				1.86 (1.11–3.14)

SLAM, South London and Maudsley NHS Foundation Trust; BMI, body mass index; HR, hazard ratio.

a. First date of follow period is 1 March 2020.

b. Index period is between 1 December 2019 and 1 March 2020, which is 3 months prior to the follow-up period.

## Discussion

### Summary of findings

Our findings suggest that receiving clozapine treatment is associated with increased risk of COVID-19 infection, compared with receiving any other type of antipsychotic treatment. Crude associations were attenuated but not completely explained by differences in sociodemographic factors such as age, gender and ethnicity, factors related to health conditions such as smoking status, BMI or proxies of availability of COVID testing (in-patient status or number of contacts with the SLAM services).

### Comparison with previous studies

To our knowledge, no previous research has specifically investigated the associations between infection with COVID-19 and receiving clozapine treatment, as compared with receiving treatment with other antipsychotics.

In previous research, the risk of COVID-19 infection has been reported to be associated with older age, male gender, ethnicity (having an African, Caribbean, Other Black background, Bangladeshi or Pakistani background, or Indian (if male)) and higher BMI.^24,25^ We found that older age was associated with COVID-19 infection and that infection rates were higher among Black people (compared with White people) and among people with high BMI (obese), but there were no significant associations with gender in our investigation.

### Strengths

The cohort was large and inclusive of all patients who met the inclusion criteria in a defined population. SLAM is a near-monopoly provider for all aspects of secondary mental healthcare to a defined catchment area, so the study represents an almost comprehensive coverage of patients receiving clozapine treatment living in this catchment area of 1.3 million people.

In this analysis, the CRIS database made it possible to explore the complete electronic clinical records of more than 6000 individuals who met our inclusion criteria, which gave us the statistical power to be able to analyse a relatively rare event, and adjust for a range of potential confounders.

In cohort studies, it is often impossible to be certain that the cases identified are true incident cases as opposed to prevalent cases that are identified during the study period. However, because there had been no cases of COVID-19 in SLAM at the start of the follow-up period, we can be certain that these are all incident cases of COVID-19. Furthermore, we can completely rule out reverse causation: the prescription of clozapine could not have been affected by knowledge of COVID-19 status since clozapine status was measured before any cases of COVID-19 had been diagnosed. Similarly, contact with services and in-patient status were measured before the start of the epidemic, so could not have been affected by COVID-19 status.

### Limitations

We controlled for a number of potential confounders; however, there may still be residual confounding. There is a very large effect of in-patient status on the risk of COVID-19 infection. This is likely to arise partly from a higher risk of exposure to COVID-19 in hospital settings, and largely from the policy that in-patients showing any symptoms of COVID-19 were tested, while testing in the community was less comprehensive. Controlling for in-patient status on 1 March 2020 has not annulled the significant association between clozapine and COVID-19 infection. However, we cannot rule out the possibility that clozapine-treated patients could be more likely to be tested for COVID-19, even after accounting for the differences in patient contact and in-patient status between the groups before 1 March 2020. Also, it is possible that clozapine-treated patients might be more likely to be symptomatic with COVID-19, possibly owing to a reduced immune response, and therefore more likely to be tested. Consequently, a conservative interpretation of these findings might be that people on clozapine treatment are more likely to suffer from *symptomatic* COVID-19 infection, which is itself important clinically.

During the study period, SLAM enacted a policy of attempting to discharge patients back into the community where possible, to free up in-patient capacity. We are making an assumption that the proportion of patients discharged did not differ between the clozapine-treated group and the non-clozapine-treated group, the in-person and remote patient monitoring did not differ between the two groups, and that the amount of care and monitoring before compared with during the pandemic remained proportional between groups. Other potential confounders, such as cardiovascular diseases, hypertension, respiratory diseases or metabolic side-effects such as obesity and diabetes, were not included in the study because reliable data were not available for the whole cohort.

The most recent BMI measurements for some patients in the study were from almost 15 years ago. Although this is likely to give some indication of their BMI at the time of the study, it is important to note that BMI is more likely to have been recently measured in the clozapine-treated participants owing to the increased monitoring.

The ‘current smoker’ status of smoking data was extracted on the basis of the status within the 12 months prior to the follow-up period, so some of those data were from almost a year ago. Some of the ‘past smoker’ and ‘never smoked’ data were from almost 18 years ago. Given the impact of smoking on clozapine metabolisation and clozapine plasma levels, we cannot rule out that clozapine-treated patients may be questioned more frequently about smoking and therefore have more up-to-date information regarding smoking habits.

### Implications

To our knowledge, our results are the first to suggest that people on clozapine treatment are at higher risk of infection by COVID-19.^21^ This is consistent with previous research demonstrating that people treated with clozapine have higher rates of infection and pneumonia than those on other antipsychotics and have alterations in both innate and adaptive immunity. There are also several alternative explanations for these findings, most notably the fact that clozapine-treated patients are likely to come into greater contact with services than patients on other antipsychotics and are therefore more likely to be tested if they develop symptoms. We have tried to adjust for patient contact, but, given the very large association between in-patient status and infection with COVID-19, we cannot confidently exclude the possibility that the association is explained by residual confounding.

The study is based on a relatively small number of cases, and we would not advocate any change in practice based on these findings alone. However, if the association is replicated and becomes firmly established, clinicians and patients will need to weigh up the increased risk of COVID-19 infection against the risk of psychotic relapse if clozapine is discontinued. Given that, for many patients, clozapine is the only effective antipsychotic, and with the well-established association between clozapine treatment and reduced all-cause mortality, these decisions are likely to be finely balanced and must be taken on a case-by-case basis.

Until this association is more firmly established, we would recommend that clinicians follow consensus guidelines for clozapine treatment during the COVID-19 pandemic, such as those of Siskind and colleagues^21^ and Luykx and colleagues.^26^ There should also be a focus on ensuring that clozapine-treated patients follow simple hygiene measures that can be taken to reduce the risks of COVID-19 infection, including handwashing, social distancing and the rigorous use of face masks and other personal protective equipment in clinical settings.

### Future research

As the COVID-19 pandemic progresses, we and other groups will be able to study this association in larger samples and perhaps with better control of confounding. It will also be important to establish whether, among psychiatric patients with COVID-19, those treated with clozapine are at differential risk of adverse outcomes such as hospital admission, pneumonia, treatment in intensive care or ventilation, or death.

## Data Availability

The study used clinical data held by South London and Maudsley NHS Foundation Trust. The data are not available to those outside this organisation.

## Supplementary material

For supplementary material accompanying this paper visit https://doi.org/10.1192/bjp.2020.151. click here to view supplementary material
