# Supplementary material for: Clozapine treatment and risk of COVID-19 infection: retrospective cohort study
Source: Br J Psychiatry. 2021 Jul;219(1):368–74. doi: 10.1192/bjp.2020.151 (PMC7417985; doi:10.1192/bjp.2020.151)
Supplement: Supplementary file 1 [file S0007125020001518sup001.zip › S0007125020001518sup001.docx]

Supplementary Table 1: Multivariate Cox analysis of the association between receiving clozapine treatment and infection with COVID-19 between March 1, 2020 and May 18, 2020, inclusive in the whole cohort (n=6309, 102 COVID-19 Positive Cases). There were missing values in ethnicity, smoker status, and BMI, so these variables were not included in this analysis.

| Risk Factors | Hazard Ratio (95% CI) | Hazard Ratio (95% CI) | Hazard Ratio  (95% CI) |
| --- | --- | --- | --- |
| On clozapine treatment  No  Yes | 1.00  2.78 (1.87 - 4.12) | 1.00  3.02 (2.02 - 4.51) | 1.00  1.73 (1.15 - 2.61) |
| Gender  Male  Female |  | 1.00  1.28 (0.86 - 1.90) | 1.00  1.37 (0.92 - 2.03) |
| Age  < 29 years  30 to 39 years  40 to 49 years  50 to 59 years  60 to 69 years  70 years and over |  | 1.00  1.22 (0.59 - 2.54)  0.62 (0.27 - 1.41)  1.40 (0.70 - 2.81)  1.85 (0.85 - 3.99)  2.77 (1.23 - 6.23) | 1.00  1.57 (0.75 - 3.26)  0.85 (0.37 - 1.93)  2.22 (1.10 - 4.49)  3.44 (1.58 - 7.50)  3.66 (1.62 - 8.27) |
| Inpatient on the first day of follow-up period*  No  Yes |  |  | 1.00  11.97 (7.13 - 20.08) |
| SLAM service contact over index period**  < 4 days  4 to 7 days  8 days and more |  |  | 1.00  2.58 (1.08 - 6.16)  3.58 (1.50 - 8.52) |

*: the first date of the follow period is March 1, 2020

**: index period is between December 1, 2019 and March 1, 2020, which is 3 months prior to the follow-up period
